# A novel comparison of Epstein-Barr virus with broad histological spectrum of oral squamous cell carcinoma

**DOI:** 10.12669/pjms.35.5.899

**Published:** 2019

**Authors:** Muhammad Wasif Saleem, Faraz Ahmed Baig, Naila Irum Hadi

**Affiliations:** 1 Muhammad Wasif Saleem (BDS, MPhil Trainee), Department of Pathology, Ziauddin University, 4/B, Sharah-e-Ghalib, Clifton Karachi, Pakistan; 2 Faraz Ahmed Baig (MBBS, MPhil, PhD Fellow), Department of Pathology, Ziauddin University, 4/B, Sharah-e-Ghalib, Clifton Karachi, Pakistan; 3 Prof. Dr. Naila Irum Hadi (MBBS, MPhil, PhD), Department of Pathology, Islamabad Medical & Dental College, Satra Meel, Main Murree Road, Bhara Kahu, Islamabad, Pakistan

**Keywords:** Acantholysis, Epstein-Barr virus, Immunohistochemistry, Smoking, Squamous cell carcinoma

## Abstract

**Objectives:**

To evaluate the immunohistochemical expression of Epstein-Barr virus (EBV) in broad spectrum histological subtypes of oral squamous cell carcinoma (OSCC) and to determine the relationship of EBV with clinicopathological parameters of OSCC.

**Methods:**

This cross-sectional study included 150 clinically diagnosed OSCC cases from the outpatient of Ziauddin University Hospital from March, 2017 to October, 2018. These were confirmed on histological examination and categorized into conventional squamous cell carcinoma (SCC) and rare variants. Conventional SCC was subcategorized into keratinizing (KSCC), non-keratinizing (NKSCC), and hybrid SCC (HSCC). EBV status was compared among various histological tumor entities and clinicopathological characteristics of OSCC using immunohistochemistry. Chi-square test was used to determine the association of each histological subtype with EBV status with P-value <0.05 considered as statistically significant.

**Results:**

Conventional tumor was the most frequent squamous cell carcinoma (n=126; 84%). A significant statistical link of EBV infection was observed with rare histological tumors exhibiting acantholysis (P=0.01), as well as tumors involving buccal mucosa (P=0.03), and habitual smokers (P=0.001).

**Conclusions:**

In this study, acantholytic tumor, a rare histological subtype of OSCC, tended to be EBV related. Moreover, OSCC cases bearing EBV infection were more likely smokers favoring buccal mucosa as primary anatomical site for oral cancer.

## INTRODUCTION

Oral squamous cell carcinoma (OSCC) accounts for 90% of all cancers arising from the oral cavity.^1^ Increase in incidence and mortality has been observed over the last decade, which is more pronounced in developing countries like India, Pakistan and Bangladesh, with a significant inverse correlation with human development index (HDI), further straining their resources.^2^ This high burden of disease is mainly contributed by the chronic use of smoking, chewable tobacco, pan, gutka, betel quid and naswar, which are commonly consumed items in developing countries.^3^,^4^ A well-established role of Human Papilloma virus (HPV) in oral carcinogenesis has been previously documented.^5^ In addition to that, the link of Epstein Barr virus (EBV), an oncogenic virus with a possible role in development of OSCC, has also been hypothesized in literature.^5^

Histologically, OSCC exhibits atypical squamous epithelial cells with variable degree of maturation, intercellular bridges and keratin pearl formation.^6^ The degree of squamous maturation may differ among cases which primarily characterize conventional tumors (Conventional Squamous Cell Carcinoma; CSCC) into keratinizing (CKSCC) and non-keratinizing (CNKSCC) entities, where the latter entity represents lack of mature squamous differentiation.^7^ Additionally, a hybrid (HSCC) variant depicting a mixed pattern of squamous maturation and an intermediate prognosis between CKSCC and NKSCC has been reported.^8^ More than 80% cases of OSCC encountered in clinical practice demonstrate conventional morphology, however other rare subtypes of squamous cell carcinoma also exist. These subtypes constitute 10-15% of all OSCC cases.^9^

In terms of histopathology, a rare subtype can only be classified, if most of the tumor volume reflects representative morphological pattern, however acantholytic and verrucous subtypes may be characterized on focal differentiation.^7^,^8^ The rare histological variants include papillary (PSCC), verrucous (VSCC), acantholytic, lymphoepithelial, adenosquamous (ASCC) and basaloid squamous cell carcinoma (BSCC), respectively.^7^-^10^

Although, unique morphological feature, referred to as cytopathic effect, is specific to certain viruses, the association of viral infections with diverse histological variants of OSCC is not well described. Interestingly, the link of HPV infection has been previously hypothesized with non-keratinizing and hybrid conventional entities,^7^,^8^ however the relationship of another virus with a possible role in oral carcinogenesis, such as EBV remains to be elucidated. The present study was designed first to evaluate the expression of EBV in broad spectrum histological subtypes of OSCC, and second to determine the relationship of EBV with clinicopathological parameters of OSCC.

## METHODS

Biopsy confirmed OSCC cases (n=150) were recruited after written informed consent from all the study participants with an effort to maintain the confidentiality of their personal details. This cross sectional study was conducted from March, 2017 to October, 2018, in accordance with Helsinki declaration with approval from the Ethics Review Committee of Ziauddin University and Hospitals, Karachi, Pakistan.

Clinically diagnosed OSCC patients of both sexes and all age groups were included while patients on antiviral therapy, with other malignant tumors, and with metastatic tumors to the oral cavity were excluded from this study. Approximately 4 µm thick sections were cut from formalin-fixed paraffin-embedded biopsy blocks and stained with Hematoxylin and Eosin (H & E) for evaluation under light microscope. WHO characterization criteria was followed for classification of tumor subtypes.^9^-^11^ Briefly, among the three conventional tumors (CSCC), keratinizing (CKSCC) entity was described by areas of mature squamous cells, whereas non-keratinizing (NKSCC) lacked this characteristic. Hybrid tumor contained areas of focal squamous maturation throughout the tumor. Lymphoepithelial, papillary and basaloid subtypes showed dense lymphocytic infiltrate, papillary growth and palisading, respectively. Whereas, adenosquamous variant exhibited focal glandular differentiation and verrucous subtype showed broad bulbous pushing rete ridges with parakeratosis. Focal areas of acantholysis or pseudoglandular features were also appreciated within some tumors characterizing them as distinct histological variants.

The EBV status was evaluated using light microscopy by all the three authors (NIH, FAB, MWS). For immunohistochemistry, 4 µm thick tissue section from formalin-fixed paraffin-embedded tissue blocks were used and stained for EBV (Cell marque Clone MRQ-47) following manufacturer’s protocol which is briefed as follows: Deparaffinization in xylene was followed by multiple steps of hydration with alcohol and normal saline. After 10 minutes of incubation in hydrogen peroxide to block endogenous peroxidase, slides were treated with Ficin and incubated with the monoclonal mouse EBV antibody at 1:100 dilution overnight at 2-8°C. The slides were then incubated with a biotinylated anti-rabbit secondary antibody (1:100 dilution) for 30 minutes at room temperature.

The slides were parmounted with cover slip and bound antibody was detected for EBV. The tissue sections were considered positive by cytoplasmic staining of the tumor cells observed under light microscopy. To authenticate the reaction, slides harboring Hodgkin lymphoma with known levels of EBV expression were used as positive control.

Statistical analyses were performed using Statistical Package for the Social Sciences (SPSS) version 20. To determine the association of each histological subtype with EBV status, cross tabulation analyses were performed using chi-square test. P-value <0.05 was considered statistically significant.

## RESULTS

Overall, 34 out of 150 biopsy confirmed OSCC showed immunohistochemical expression of EBV protein. Table-I demonstrates clinicopathological characteristics of all study subjects included in the present research.

**Table I:** Clinicopathological and demographic parameters of all study subjects.

	Total (n=150)	%	EBV positive (n=34)	%	EBV negative (n=116)	%	p value*
***Sex***							
Male	115	76.6	33	97	82	70.6	NA^†^
Female	35	23.4	01	03	34	29.4	NA^†^
Mean age	49.5±5		55±5		48.5±5		
***Habit***							
Smoking	40	26.6	20	58.8	20	17.2	0.001**
Alcohol	09	06	02	5.8	07	6.03	1.00
Gutka	27	18	04	11.7	23	19.8	0.32
Betel nut	31	20.6	03	8.8	28	24.1	0.06
Naswar	14	9.3	01	2.9	13	11.2	0.19
Pan	29	19.3	04	11.7	25	21.5	0.32
***Site***							
Buccal Mucosa	68	45.3	21	61.7	47	40.5	0.03**
Floor of the mouth	25	16.6	07	20.5	18	15.5	0.60
Tongue	20	13.3	02	5.8	18	15.5	0.24
Lip	13	8.6	02	5.8	11	9.4	0.73
Maxilla	17	11.3	01	2.9	16	13.7	0.12
Mandible	07	4.6	01	2.9	06	5.1	1.00
***Grades***							
Well Differentiated	54	36	13	38.2	41	35.3	0.84
Moderately Differentiated	79	52.6	17	50	62	53.4	0.85
Poorly Differentiated	17	11.3	04	11.7	13	11.2	1.00

* Chi-square analysis.

** Represents significant association.

† Not applicable/ assessable.

Majority of OSCC cases were males, which also constituted the major portion of EBV positive cases, whereas only 01 female case showed expression of EBV protein. The mean age for all study subjects was observed as 49.5 ± 5 years, whereas 55 ± 5 years for EBV positive cases.

For anatomical locations, tumors arising from the buccal mucosa were most frequent, followed by floor of the mouth and lip. The same order of anatomical location was observed for EBV infected cases as well. Regarding habits as risk factors for OSCC, we observed that smoking was on top of the list in cases bearing EBV infection as well as those without infection. A strong statistical association of smoking with EBV infection was observed (p < 0.05). Similar finding was recorded for buccal mucosa (P<0.05). Most of the tumors included in the study were moderately differentiated and this trend was also observed for distribution of EBV infection (Table-I).

While characterizing tumor subtypes, the highest number of cases exhibited conventional morphology (Table-II). These were further sub categorized into KSCC (30%), NKSCC (38.6%) and HSCC (15.3%) respectively, with NKSCC exhibiting the highest frequency of EBV infection. Among rare variants, adenosquamous carcinoma was most frequent, followed by lymphoepethelial, verrucous, acantholytic, basaloid and papillary carcinoma, respectively. For EBV, all acantholytic tumors were positive, however the distribution of infection was uniform between verrucous and adenosquamous as well as between basaloid and lymphocytic variants. A strong statistical association of EBV infection with acantholytic tumors was determined, however none of the other OSCC histological entity showed any statistical difference with the virus (Table-II).

**Table II:** Comparison of Histological variants of OSCC with EBV expression.

Histological type	Total cases (n=150) n (%)	EBV + ve (n=34) n (%)	EBV - ve (n=116) n (%)	P-value*
***Conventional***				
Keratinizing squamous cell carcinoma (KSCC)	45 (30)	08 (17.7)	37 (82.3)	0.349
Non-Keratinizing squamous cell carcinoma (NKSCC)	58 (38.6)	13 (22.4)	45 (77.6)	0.953
Hybrid squamous cell carcinoma (HSCC)	23 (15.3)	05 (21.7)	18 (78.3)	0.908
***Variants***				
Verrucous	04 (2.6)	02 (50)	02 (50)	0.221
Adeno-squamous	09 (6)	02 (22.2)	07 (77.8)	1.00
Acantholytic	02 (1.3)	02 (100)	00 (0.0)	0.01**
Basaloid	02 (1.3)	01 (50)	01 (50)	0.403
Lymphoepithelial	06 (4)	01 (16.6)	05 (83.4)	1.00
Papillary	01 (0.6)	00 (0.0)	01 (100)	1.00

* Chi-square analysis,

** Represents significant association. EBV + ve – Epstein-Barr virus positive, EBV – ve – Epstein-Barr virus negative.

**Fig.1:**
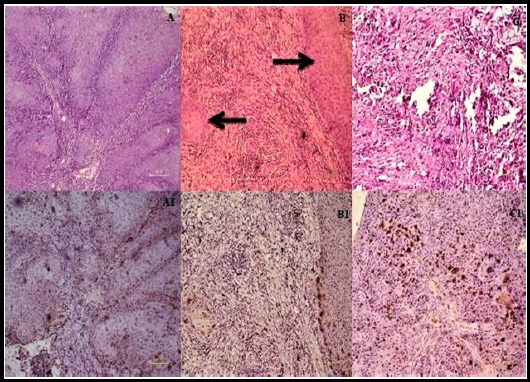
Histological and immunohistochemical characterization of Conventional squamous cell carcinoma (A) Representative section of keratinizing SCC composed entirely of mature squamous cell (A1) EBV cytoplasmic and membrane expression in KSCC. (B) Representative case of hybrid SCC exhibiting atypical non-maturing cell sheets with focal squamous maturation and pushing borders (B1) EBV expression in hybrid SCC. (C) Representative case of non-keratinizing squamous cell carcinoma exhibiting non-maturing tumor cells arranged in sheets (C1) EBV expression in non-keratinizing squamous cell carcinoma. [A to C, Hematoxylin and eosin staining, A1 to C1 Immunohistochemistry staining, with original magnification: x200 respectively].

**Fig.2:**
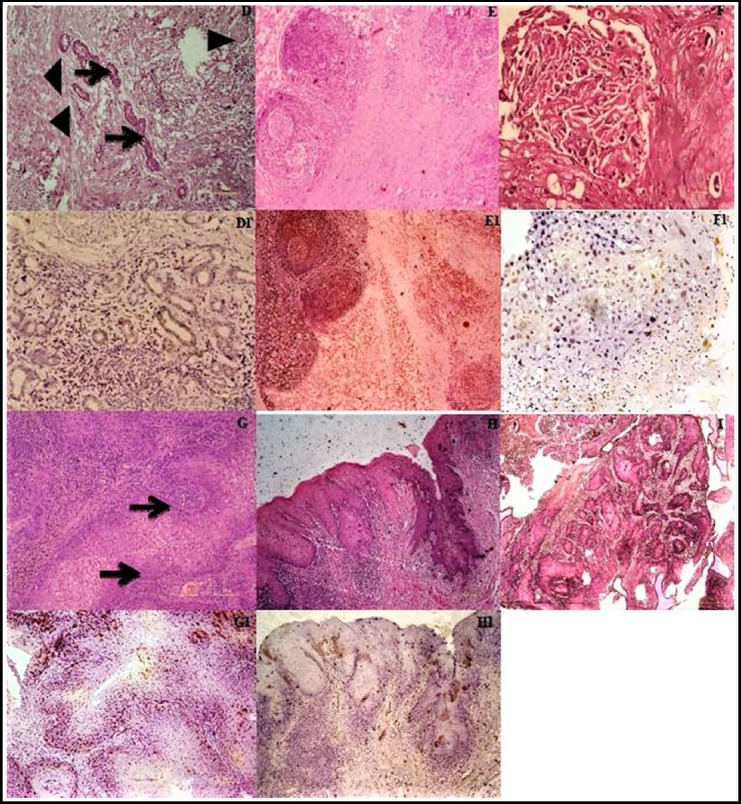
Histological and immunohistochemical characterization of variants (D) Representative case of adenosquamous cell carcinoma showing multiple atypical glands (arrows) along with neoplastic squamous components (arrow heads). (D1) EBV expression in adenosquamous cell carcinoma. (E) Representative case of lymphoepithelial SCC exhibiting nest of tumor cells in a dense mononuclear inflammatory background. (E1) EBV expression in lymphoepithelial SCC. (F) Representative case of acantholytic SCC showing pseudo-glandular architecture with focal acantholysis within tumor nest. (F1) EBV expression in acantholytic SCC. (G) Representative case of basaloid SCC showing palisading (arrow) along with atypical squamous cells. (G1) EBV expression in basaloid SCC. (H) Representative case of verrucous SCC showing well differentiated squamous cell carcinoma with marked keratinization. It has pushing borders and chronic inflammatory response. (H1) EBV expression in verrucous SCC. (I) Representative case of papillary SCC showing nests of tumor cells arranged in papillary fashion. [D to I, Hematoxylin and eosin staining, D1 to H1, Immunohistochemistry staining, with original magnification: x200 respectively].

Fig. 1 & 2 demonstrate conventional and rare subtypes of OSCC, accompanied by representative EBV positive immunohistochemical sections.

## DISCUSSION

Worldwide, the incidence of oral squamous cell carcinoma (OSCC) differs widely, while the prevalence is reported to be the highest in Indo-Pak subcontinent.^11^ Multiple histopathological subtypes of OSCC are described in literature.^7^-^10^ According to indexed literature, the major risk factor for development of OSCC in our region is HPV infection secondary to chronic use of tobacco, pan, gutka, betel nut and naswar.^12^,^13^

Viral induced cellular alterations are the hallmarks of some infections and proved useful in screening out biopsy specimens harboring infections.^9^ Inspired by this idea, few studies have screened HPV among limited histological spectrum of OSCC and documented some interesting findings.^7^,^8^ Carrying this idea forward, we took a novel initiative to determine the relationship of EBV with various histological subtypes of OSCC in a larger series of samples. Previously, this virus (i.e. EBV) has been reported in OSCC specimen as an established cause of tumors originating in head and neck vicinity.^7^

We determined EBV protein expression in both conventional and rare morphological types of OSCC. However, we failed to observe statistical difference with conventional tumors as well as most rare subtypes encountered in our analysis. Among conventional tumors, NKSCC histology indicated sequentially higher frequencies of EBV positivity compared to KSCC and HSCC.^8^ Strong association of EBV with NKSCC was previously reported in nasopharyngeal carcinoma.^14^ To the best of our knowledge, this is the first study to investigate the relationship of EBV with diverse histological subtypes of OSCC worldwide. Hence, no data regarding EBV and OSCC tumor variants is available for comparison.

Interestingly, the only histological entity reflecting a strong statistical link was acantholytic variant. This finding could be explained by the fact that acantholysis is a well-recognized cytopathic effect linked with viral infection caused by Herpes simplex virus, which belonged to the same family as EBV.^15^ However, possibility of another virus causing this feature cannot be ruled out. Therefore, we recommend further in-depth studies including more samples of this particular variant to confirm this link.

For histopathology subtypes, conventional squamous cell carcinoma was the most common subtype encountered in the present study (84%), and this finding is almost universal in all local and international studies.^7^,^8^,^11^ However, the frequency of rare variants differing across studies generally ranged from 1% to 15% without any subtype preference.^9^ In the present research, we encountered six different rare entities in our series of OSCC cases. Most cases were adenosquamous (6%) whereas, least commonly observed variant was papillary (0.6%). Previously, Thompson et al reported similar frequency of papillary OSCC.^15^

Among other rare variants, Neville et al reported that verrucous carcinoma accounts for 1% to 10% of all OSCCs.^16^ In present research, the frequency of this tumor type was within the reported range. Regarding basaloid squamous cell carcinoma (BSCC), Epstein-Barr virus has shown strong association with nasopharyngeal basaloid squamous cell carcinoma (BSCC), but this has not been reported in BSCC of other head and neck sites.^17^ While one out of two BSCC cases was positive for EBV in the present study, our insignificant statistical findings correspond to documented literature.^17^ Also, we came across six cases of lymphoepithelial tumors, which constitute 4% of total OSCC cases. This figure is in line with reported data of this tumor.^7^

As far as our secondary objective is concerned, an overall insignificant link of EBV with OSCC was observed. This finding is contrary to the regional study conducted by Acaharya and co-workers.^5^ Furthermore, our clinical data shows male dominance, a tumor preference for younger age groups and predilection for involvement of buccal mucosa, findings which are consistent with regional studies.^19^ We believe that male dominance and early onset of disease could be reflected by bad habits leading to oral cancer, being more common among men of younger age group.

Both local as well as international studies highlight tobacco smoking and alcohol as most common risk factors for development of OSCC.^5^ Interestingly, alcohol was not reported among habits in our cases mainly due to social and religious barriers, however smoking was the leading habit observed in our analysis. This was also supported by strong statistical difference with EBV infection. The positive effect of tobacco on HPV infection has been described in oral cancer.^18^ We suggest that our finding may have contributed similar effect on EBV, however the role of EBV behind progression of oral cancer in tobacco users remains to be determined.

Regarding the site of involvement of oral cavity, occurrence of OSCC was found considerably higher on buccal mucosa.^19^ In the present study, the statistical relation between this site and EBV infection was found to be significant. Contrary to that, most studies have reported tongue as chief anatomical location,^20^ however the link with EBV infection has not been investigated. We suggest that the reported statistical difference between buccal mucosa and EBV infection may be related to specific receptor C3D, exclusively expressed on surface of keratinized squamous epithelium lining the buccal mucosa. This receptor mediated pooling of virus has been previously established in nasopharyngeal carcinoma.^21^

Degree of tumor differentiation is another aspect of present histopathological analysis. Previously, Raab-Traub et al. and Pearson et al. indicated presence of EBV DNA in histological subtypes of nasopharyngeal carcinoma.^22^,^23^ In contrast, present study detected EBV protein in OSCC tissue and found no association of EBV infection with any pattern of differentiation. The difference in results can be attributed to the differences in sample type, analytical technique used and type of neoplasm.

### Limitations of the study

Firstly, a series of large cohort may have proven more useful in investigating the link of EBV with oral squamous cell carcinoma and its parameters. Secondly, more robust techniques such as polymerase chain reaction (PCR) and fluorescent in situ hybridization (FISH) could have provided us with more sensitive tools in determining our outcomes. Thirdly, the positive link of EBV with acantholytic tumor variant could be biased by presence of another virus involved in development of this morphological characteristic.

## CONCLUSIONS

In our region, most cases of oral squamous cell carcinoma (OSCC) are reported during fifth decade of life, with male preponderance. Buccal mucosa is the most commonly affected site. Histologically, conventional squamous cell carcinoma is the most common subtype, while moderately differentiated OSCC is the most frequent presentation. We suggest that tumors presenting acantholysis are more likely to be associated with EBV infection, a hypothesis that needs to be further validated.
